# More Than Shelter: Housing for Urban Maternal and Infant Health

**DOI:** 10.3390/ijerph18073331

**Published:** 2021-03-24

**Authors:** Jason Reece

**Affiliations:** Knowlton School of Architecture, Faculty Affiliate, The Kirwan Institute for the Study of Race & Ethnicity, The Ohio State University, 275 West Woodruff Avenue, Columbus, OH 43210, USA; reece.35@osu.edu

**Keywords:** housing, infant health, maternal health, life course, social determinants, children

## Abstract

Housing quality, stability, and affordability have a direct relationship to socioemotional and physical health. Both city planning and public health have long recognized the role of housing in health, but the complexity of this relationship in regard to infant and maternal health is less understood. Focusing on literature specifically relevant to U.S. metropolitan areas, I conduct a multidisciplinary literature review to understand the influence of housing factors and interventions that impact infant and maternal health. The paper seeks to achieve three primary goals. First, to identify the primary “pathways” by which housing influences infant and maternal health. Second, the review focuses on the role and influence of historical housing discrimination on maternal health outcomes. Third, the review identifies emergent practice-based housing interventions in planning and public health practice to support infant and maternal health. The literature suggests that the impact of housing on infant health is complex, multifaceted, and intergenerational. Historical housing discrimination also directly impacts contemporary infant and maternal health outcomes. Policy interventions to support infant health through housing are just emerging but demonstrate promising outcomes. Structural barriers to housing affordability in the United States will require new resources to foster greater collaboration between the housing and the health sectors.

## 1. Introduction

Housing is as a critical opportunity structure in society, providing far more than just shelter. Safe, stable, and affordable housing in good neighborhoods is essential to family wellbeing. Research has demonstrated the beneficial impact of housing quality and stability on outcomes ranging from economic, to educational or even psychological. Housing also has a direct relationship to aspects of socioemotional and physical health. The relationship between housing and health has long been reflected in research and policy, but the complexity of this relationship and the depth of its impact is still being understood. The literature suggests that the impact of housing on health is complex, multifaceted, and intergenerational. The following literature review seeks to understand the deep and complex relationship between urban housing challenges and health, with an emphasis on infant and maternal health.

Infant and maternal health outcomes are longstanding benchmarks for the overall wellbeing of society. Studies have consistently found very high correlation between infant mortality rates and measures of overall societal wellbeing, such as the Human Development Index (HDI). The HDI was able to predict 85% to 92% of infant mortality variation between nations [1]. Globally, infant health outcomes have improved dramatically in recent decades. Global infant mortality rates have dropped from 64.5 infant deaths per 1000 live births in 1990 to 28.2 infant deaths in 2019 [2].

### 1.1. The U.S. Paradox in Infant and Maternal Health

The United States presents an interesting case to understand the influence of inequality and inequity in maternal health within a high wealth nation. Similar to international trends, U.S. infant mortality rates have declined in the past three decades, from 9.4 infant deaths per 1000 live births in 1990 to 5.6 infant deaths in 2019 [2]. Although the United States mirrors international trends in declining infant mortality, it ranks poorly among high wealth nations and contains extreme racial and spatial disparities in infant health. From an international perspective, infant health outcomes vary substantially among nations, but the United States (as a wealthier Organisation for Economic Cooperation and Development (OECD nation) is an outlier, with infant mortality rates nearly twice the rate of OECD nations as a whole and ranking 33rd out of 36 OECD nations ranked by infant mortality rate [3].

Analysis by the U.S. Centers for Disease Control found that much of the difference between U.S. and European infant mortality rates was associated with the higher prevalence of preterm birth in the United States [4]. Preterm birth is the most common contributor to infant mortality in highly industrialized nations and is causally linked with chronic and acute psychosocial stressors facing expectant mothers [5] and political determinants of health [6].

Within the context of the United States, substantial variation exists in infant mortality rates among socioeconomic groups and racial and ethnic populations and across geography. Infant mortality rates vary among U.S. States, with the highest rates occurring in the Southeastern United States and in Ohio and West Virginia. These states reported infant mortality rates corresponding to more than 6.9 deaths per 1000 live births in 2018, and internationally rates in these states were 75% higher than the average for OECD nations (3.9) [3]. Generally, infant mortality rates are higher in U.S. nonmetropolitan (rural) counties, particularly in the rural areas within the Southeastern United States [7], although county-wide infant mortality rates in metropolitan counties often mask large geographic disparities by neighborhood, particularly in high-poverty metropolitan neighborhoods [8].

**Figure 1:**
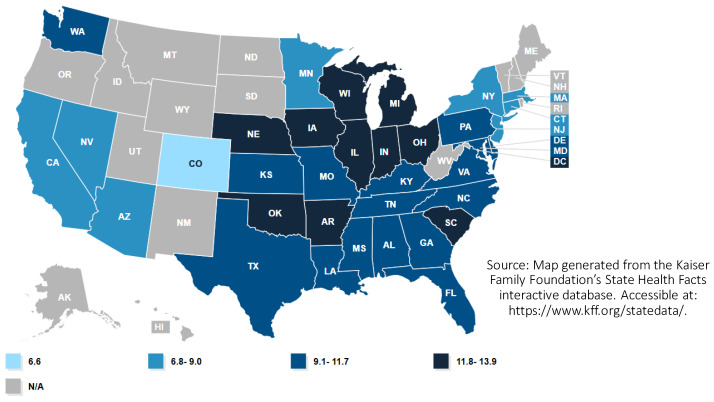
Black infant mortality rate by state (infant deaths per 1000 live births) in the United States in 2018 [15].

Beyond geography, racial and ethnic groups have the largest disparities in infant mortality in the U.S. context. Nationally, the Black infant mortality rate in 2018 (10.8 per 1000 live births) was more than double the rate for White infants (4.6), and the Native American infant mortality rate (8.2) was nearly double the rate for White infants [9]. Alarming disparities exist in maternal mortality in the United States, with maternal mortality rates for Black mothers (40.8 mortality incidences per 100,000 births) being more than 320% the rate experienced by White mothers (12.7). Mortality rates for Native American mothers (29.7) were nearly 240% higher than rates for White mothers [9]. Infant mortality rates experienced by the Black community in the United States are persistently high across geographic contexts, with the disparity in infant mortality rates between Black and White populations consistent across both metro and non-metro counties [10]. Among U.S. States, the Black infant mortality rate is highest in the Great Lakes region and the South [11] (Figure 1). Within the post-industrial Great Lakes states, Black infant mortality is highest in highly segregated and higher poverty metropolitan neighborhoods.

Racial disparities in maternal health outcomes in the United States are not the result of inherent biological differences but are a direct outcome of racism experienced by women of color. Racism and social disadvantage are intertwined structural determinants of health in the United States, with Black mothers more likely to receive repetitive prenatal stress from systemic discrimination [12]. State-level indicators of structural racism in employment and education were found to be directly associated with differences in infant mortality rates for Black but not White women in the United States [13].

Analysis of structural conditions for the Black community at the metropolitan level measured by the Birth Equity Index (ranking metropolitan areas with the worst social, economic, and environmental conditions) also found a strong association between these structural determinants of health and Black infant mortality [14]. As described by Wallace et al. [14], “The experiences of Black women in their homes, neighborhoods, and health care centers and the contexts in which they live may individually and collectively contribute to persistent racial inequity in infant mortality” (p. 1). Wallace et al. found high levels of residential (housing) segregation to be associated with the larger disparities in Black/White infant mortality rates at the metropolitan scale [14].

### 1.2. Housing as a Social Determinant of Health and Housing as a Vaccine

Housing is an important and influential social determinant of health, leading many in the field of public health to call for housing interventions as critical for addressing health disparities. Among numerous research studies that emphasize socioeconomic conditions as health determinants, housing has been identified as a primary social determinant affecting the health status of individuals, leading scholars to conceptualize stable safe housing as a critical social determinant “vaccine” for promoting health [16]. This review focuses on contributing clarity to our understanding of housing and infant/maternal health through an exploration of the existing literature in the context of U.S. metropolitan regions. Focusing on housing conditions in U.S. metropolitan areas, I conduct a multidisciplinary literature review to explore the relationship between housing and infant and maternal health. I explore the relationship through three primary perspectives. Section 2 synthesizes the literature to present the primary pathways by which housing influences infant and maternal health. Section 3 focuses on the role and influence of historical housing discrimination in the context of maternal health in metropolitan areas of the United States. Section 4 identifies emerging practice-based housing interventions in planning and public health practice to support infant and maternal health. I conclude by discussing structural and resource barriers which inhibit housing and health collaborations.

## 2. Pathways of Influence: Housing as a Contemporary Social Determinant of Infant and Maternal Health

**Figure 2:**
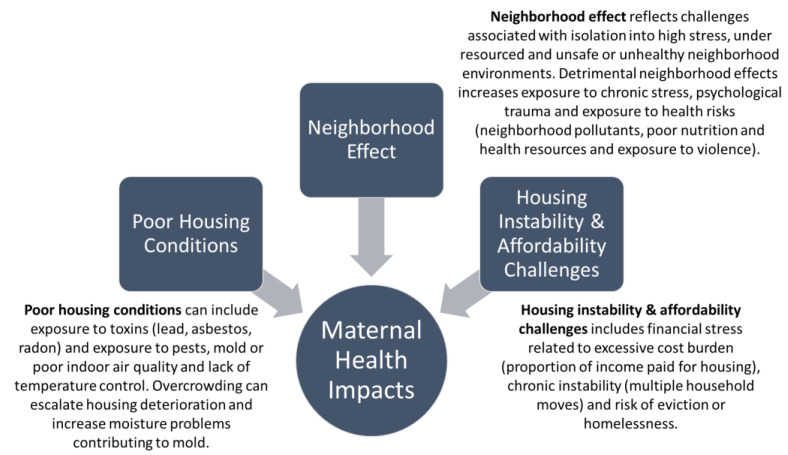
Housing ‘pathways’ that influence infant and maternal health outcomes.

Housing is a multidimensional contemporary risk factor influencing health. Building upon the literature, I categorize three primary pathways by which housing directly influences infant and maternal health. These pathways include housing condition and habitability, neighborhood effect (related to housing’s location), and housing stability/affordability (Figure 2). These three factors also play a profound role in affecting infant and maternal health outcomes.

### 2.1. Habitability and Housing Condition

Housing condition is a long-recognized factor affecting health [17]. Exposure to toxins (lead, asbestos, radon), infestation of pests, and mold and poor indoor air quality have long been linked to health risks, particularly for children [18,19]. Degraded housing conditions are also explicitly identified as being influential in infant and maternal health outcomes. Housing condition is considered a risk factor for sleep-related infant injury or death. Dangerous housing conditions are more likely to be associated with unsafe sleeping environments for infants [20,21]. Research also suggests that housing conditions alone are a risk factor even when safe sleep environments are provided. Infants are more at risk for sleep-related death even when a crib or bassinet is present in homes with poor housing conditions, due to factors such as crowding, unstable temperatures, and vermin infestation [22].

### 2.2. Segregation and Neighborhood Effect

The neighborhood effect is associated with neighborhood environments that are detrimental due to crime, resource deprivation, blight, and degraded social capital. Decades of literature have identified the profound impact of neighborhood conditions on outcomes for those living in the most disadvantaged or “low-opportunity” neighborhoods [23,24,25]. These neighborhoods are also more likely to be highly segregated areas, along dimensions of race and class. More recently, research has focused explicitly on the relationship between neighborhood conditions and health [26,27]. With respect to infant and maternal health, the literature finds a direct relationship between neighborhood conditions, segregation or isolation, and poor maternal health outcomes.

A number of research studies conclude that racially segregated neighborhoods provide worse health outcomes for infants and mothers. Racial residential segregation can cause neighborhood deprivation, poverty, and crime. In racially segregated areas, the availability of essential resources can be limited. These resources include housing, employment opportunities, access to healthy food, quality education, and quality healthcare facilities [28]. Neighborhoods that are racially segregated tend to have a higher prevalence of deprivation. Neighborhoods with high rates of economic deprivation were found to have a strong association with preterm birth [29].

Research has identified four pathways that link individual-level stressors and preterm delivery in racially segregated neighborhoods. The first is adverse health behaviors such as smoking and poor nutrition. The second is psychological factors such as lack of social support and depression. The third pathway is through stress hormones, which can initiate early labor. Lastly, women living in deprived neighborhoods can have a depressed immune system, which can lead to an increased susceptibility to infection [30]. Neighborhood segregation also correlates with increased exposure to environmental toxins in communities that have experienced environmental racism. Racially and economically segregated neighborhoods are more likely to have an overrepresentation of various environmental risks, ranging from lead exposure to exposure to poor air quality [31,32].

In poor neighborhoods, racial residential segregation has produced higher concentrations of poverty and individual-level stressors in the Black population compared to the poor White population, who is less likely to live in areas of concentrated poverty [33]. In hypersegregated metropolitan areas, it was found that babies born to black women were 50% more likely to be born prematurely compared with babies born to white women. Moreover, black infants in hypersegregated areas had significantly worse preterm birth rates when compared to black infants in non-hypersegregated areas [30].

Neighborhood crime has been shown to directly influence health outcomes. Moreover, fear of crime is directly associated with poor health outcomes and neighborhood deprivation. High rates of neighborhood crime can directly affect preterm birth because they can produce increased levels of stress in mothers, along with a lack of physical activity [29]. Neighborhoods with higher rates of violent crime may have low economic opportunity, infrastructure decay, and lack of social support, which are a direct source of chronic stress for expectant mothers [34]. Social support can be a protective factor for health, but social support systems may be impaired by isolation.

Finally, another neighborhood-level influence that relates to infant and maternal health is the presence of physical incivilities such as vacant housing or blight, litter, and graffiti. Research found that neighborhoods with high levels of crime and physical incivilities were associated with higher rates of smoking and inadequate weight gain for white and black women. Both of these factors are associated with preterm and low-birth-weight births [35]. Chronic neighborhood stress, depression, lack of social support, and behavioral health challenges are interrelated. For example, a behavioral health outcome, such as smoking, is a response to the high degree of stress and the higher rates of depression found in deprived neighborhood environments [36,37].

### 2.3. Housing Stability/Affordability

Housing instability and lack of affordability is another dimension of housing which can contribute to poor infant and maternal health. Housing instability is a significant predictor of lower birth weight and a corollary of educational disparities, lack of financial support, and food insecurity [38]. Housing instability is associated with screening positive for depression and anxiety among mothers, regardless of other social stressors [39]. Previous literature documents the various forms of housing instability that impact maternal health. Housing instability is represented by several different events: foreclosure, transience, eviction, and homelessness.

Housing challenges facing families with young children are most pronounced for lower income renter households. Sandel et al. (2018) assessed housing risks for more than 22,000 lower income renters who were caregivers for young children and found 86% of caregivers had experienced a form of housing deprivation and 34% had experienced serious housing instability, including being behind on housing payments (rent), experiencing multiple housing moves, or experiencing homelessness. Households who had experienced these three forms of serious housing deprivation were more likely to experience poor caregiver health, maternal depressive symptoms, and poor child health [40].

Foreclosure has been associated with impaired mental health, which can directly affect birth outcomes [41]. Losing a home to foreclosure induces an extreme level of stress to the individual, representing an acute form of trauma. Women who have experienced foreclosure have higher risk of depressive symptomatology. For new mothers, the experience of foreclosure nearly doubled the risk of severe depressive symptoms [39]. Transience (multiple housing movements), as a form of housing instability, also negatively impacts birth outcomes [38,39]. Research has found that multiple moves preceding birth are associated with lower birth weight and poor maternal mental health.

Eviction and homelessness, which are two of the most disastrous events in terms of housing instability, are directly linked to negative health outcome for mothers. Eviction was a significant traumatizing factor for low-income urban mothers. Mothers who were evicted experienced higher rates of material hardship, depression, worse health for themselves and their children, and high parental stress. The eviction effect lasted for more than two years with respect to depression and material hardship [42].

Homelessness is bidirectionally linked to infant and maternal health concerns. Maternal health challenges escalate the risk of homelessness. Robust associations exist between postpartum maternal depression and risk for homelessness [43]. Mothers and infants who experience homelessness are more likely to experience birth complications, mental health challenges, and other acute and chronic health impairments [43,44]. The severity of homelessness predicted low birth weight and preterm births and outweighed other risk factors [45,46]. Sandel et al.’s study (2018) of 20,571 caregivers of young children found children who experienced either prenatal or postnatal homelessness had the highest risk for postneonatal hospitalization, poor child health, and development challenges [40].

Cutts et al. (2018) assessed the relationship between health outcomes for nearly 10,000 mothers and housing conditions. Their analysis found the experience of homelessness during infancy to be associated with both poor infant health and poor physical and mental health for mothers [47]. The experience of homelessness for infants and young children produces an extreme form of stress and deprivation at a critical time for brain development, contributing to developmental delays in “cognitive, socioemotional and motor ability” [47] (p. 121).

The implication of this larger body of research suggests that women who have suffered housing instability prior to or during pregnancy had much higher levels of stress and various other mental health complications. Housing not only provides physical shelter and safety but also is a psychological form of refuge, and its destabilization can lead to severe mental health consequences. Young children who experience housing deprivation are exposed to a form of toxic stress that occurs at a critical moment of development, contributing to a variety of long-term health consequences.

## 3. Pathways of Influence: Historical Housing Discrimination as an Intergenerational Health Stressor

Social determinants of health, particularly those impacting infant and maternal health, are not limited to just contemporary issues. The Life Course Theory posits that the long-term impacts of accumulated stress can shape contemporary maternal health outcomes [48,49]. Forms of historical and contemporary structural and institutional racism have persisted in the United States, contributing directly to racial disparities in maternal health today. As described by the National Academies of Sciences, Engineering, and Medicine, these forms of historical trauma play a role in community resiliency and health risk today.

“Historical trauma, a collective complex trauma inflicted on a group of people who share a specific group identity or affiliation, manifests from the past treatment of certain racial and ethnic groups…This is another form of structural (i.e., systemic) racism that continues to shape the opportunities, risks, and health outcomes of these populations today”

[50] (p. 112).

### 3.1. Historical Housing Discrimination as a Form of Historical Trauma

Various types of housing discrimination throughout the 20th century are a form of historical trauma. An extensive infrastructure of policies enforcing housing discrimination proliferated in U.S. cities throughout much of the 20th century. These factors included racial zoning, expulsive zoning, exclusionary zoning, enforcement of racially restrictive covenants, redlining, urban renewal, de facto segregation in public housing, and reverse redlining or predatory lending [51,52,53,54,55,56,57]. Historical housing discrimination and disinvestment has been linked to long-term neighborhood blight and instability, therefore contributing to the contemporary “neighborhood effect” challenges of today in many poor urban neighborhoods.

**Figure 3:**
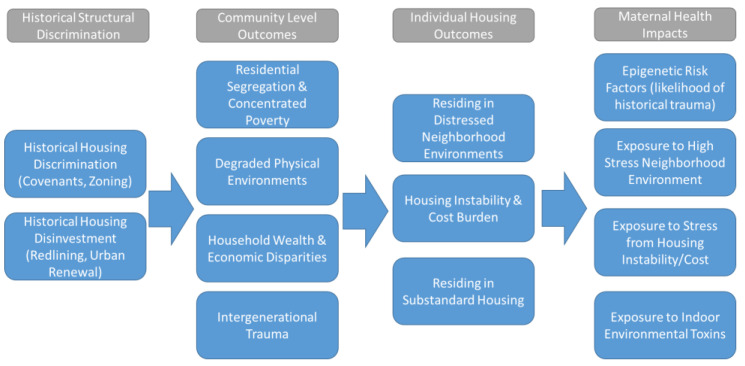
Influence of historical forms of housing discrimination on contemporary community and health outcomes.

Two primary forms of housing discrimination have impacted urban communities of color, the de jure and de facto racial, ethnic, and class-based segregation imposed on housing markets and the institutionalized disinvestment and disruption or demolition of urban communities of color. These historical phenomena were enacted through a variety of policies and practices throughout the 20th century. The result of these historical forces created several detrimental community-level outcomes in urban communities of color, which include high rates of residential segregation and concentrated poverty, degraded physical (or built) environments within urban communities of color, household wealth and economic disparities, and the increased likelihood of intergenerational trauma within urban communities of color (Figure 3).

### 3.2. Health Impacts of Historical Housing Discrimination

These community outcomes set the stage for the contemporary housing challenges found today in segregated lower income neighborhoods. These include distressed or degraded neighborhood environments, a higher likelihood of residing in substandard housing, and housing instability or affordability (cost burden) challenges. These historical factors and the resulting contemporary housing challenges thus create extreme levels of health risk for mothers and infants. Mothers in these communities are more likely to be experiencing epigenetic risk factors (from historical trauma), are facing greater exposure to high-stress neighborhood environments, are more likely to be exposed to stress related to housing instability and cost, and finally are more likely to be living in housing with greater indoor environmental risks and hazards (lead, pests, etc.).

Beck et al. (2019) suggest that historical forms of housing discrimination and today’s racial disparities in relation to preterm birth are deeply interconnected [57]. The legacy of historical and contemporary housing discrimination has resulted in three causal pathways for poor infant health outcomes, which include isolation into areas with poor and non-culturally competent health care, increased risk from exposure to toxic forms of stress for mothers, and segregation into socioeconomically disadvantaged environments for mothers, infants, and young children [58].

The historical connection between housing discrimination and infant health is supported by emerging studies exploring the relationship between patterns of “redlining” and preterm birth, such as that by Krieger et al. (2020). This research reviewed birth records for 528,000 births in New York City, assessing the relationship between contemporary preterm birth and residency within formerly redlined areas. Areas within New York City that were redlined prior to 1940 had the highest rates of preterm birth (7.3%), and residency in these areas was still significant as a predictor of higher preterm birth rates after adjusting for contemporary socio-demographic characteristics [59].

Housing and development policy has always played and continues to play a profound role in either disenfranchising or empowering urban communities of color. As a focus of health intervention, housing investment and stabilization is critical to remedying contemporary challenges but also essential to remedying the historical trauma inflicted upon urban communities of color through discriminatory housing policy.

## 4. Housing for Maternal and Infant Health: Models of Planning and Public Health Intervention

City planning and public health have focused on the intersection of housing and health since the late 19th century. The emergence of building codes and early zoning during the late 19th and early 20th century (and later slum clearance and public housing in the mid-20th century) was motivated by concerns for public health and in some cases infant mortality. Although not engaged in this literature review, the evolution of the environmental justice (EJ) movement in the 20th century was another important advancement in aligning housing and health concerns. The grassroots origins of the EJ movement were critical to policy reforms to address exposure to both indoor and neighborhood environmental toxins.

In recent decades, the enhanced emphasis by policymakers and practitioners on social determinants of health has reinvigorated collaborations which leverage housing as an infant and maternal health intervention. Although these interventions remain emergent, initial positive outcomes point to the need for further exploration of these interventions. I categorize contemporary housing efforts focused on maternal health into three primary models of intervention. These include direct housing assistance targeted toward higher risk expectant families or those with young children, housing mobility programs which seek to provide stable housing in healthy and well-resourced neighborhoods, and community wide planning or neighborhood improvement strategies.

In implementation, these strategies have been approached as isolated interventions or broader interventions which seek to integrate several of them. Interventions discussed in this review are primarily led by planning organizations, public health agencies, community-based organizations, or health care stakeholders. Although the focus of this review is on efforts led by government or nonprofit organizations, we must also acknowledge the historical and contemporary importance of community organizing, resident-led activism, participatory planning, and larger social movements, that are led by marginalized communities. These social movements are critical to building momentum for public health, environmental justice, and housing interventions [60,61].

### 4.1. Direct Targeted Housing Assistance

Targeted housing assistance efforts direct or “triage” housing resources toward higher risk expectant families or families with young children. Housing resources and services are often coordinated with other wrap-around health care and social services. Housing for health initiatives have been piloted for a variety of medically vulnerable populations (or households with high health care expenditures who are referred to as medically complex families) [62]. Targeted housing assistance can also include legal assistance to support families in assuring their housing rights and civil rights are not violated. Medical Legal Partnerships align legal aid with medically complex families who are in housing crisis and can assist in navigating landlord tenant disputes or eviction pressures [63,64].

A multi-year scan of housing for health interventions implemented between 2010 and 2015 in the United States conducted by the Urban Institute reviewed 13 programmatic interventions implemented by the State or local government [65]. The environmental scan of interventions found common themes, challenges, and opportunities across these programs. State governments were the most likely entities to enact these direct intervention programs, often utilizing Medicaid funding to align medical and supportive services. Flexible Medicaid policies which allowed accrued health care savings to be redirected to offset housing or other costs were the most effective. Interventions rarely invested directly into new housing but were more likely to focus on alignment of health care services around providing access to existing affordable housing units. The programs early evaluation outcomes were promising, but the coordination required to implement these initiatives and continued reluctance of health care institutions to invest outside of health services was an ongoing barrier to further expansion of the programs.

### 4.2. Housing Mobility Programs

Housing mobility programs align affordable housing opportunities in healthy and high-resource neighborhoods for lower income renter families with young children [66]. Mobility programs are often regarded as a form of affirmative fair housing policy that seeks to counteract patterns of racial and economic segregation and exclusion in the housing market. The largest direct affordable housing program in the nation, the Housing Choice Voucher program, allows low-income families to utilize housing throughout metropolitan areas, although research has found discriminatory barriers still restrict most voucher holders into areas with high levels of neighborhood distress [67].

Most research on the health benefits of housing mobility has been generated through either the Move To Opportunity (MTO) experimental housing program implemented in the 1990s or small scale housing mobility programs launched in response to fair housing litigation [68]. Evaluations of mobility programs have demonstrated health benefits to children and mothers, particularly related to mental health and reduced long-term medical costs, although programs have produced less robust positive health outcomes for male participants than for female participants [69]. Positive health outcomes were also sensitive to the age of children in mobility programs, with children under the age of 13 experiencing the strongest long-term health improvements. In 2020, 25 housing mobility programs were either operational or emerging throughout the United States; the U.S. Department of Housing and Urban Development has launched a $50 million competitive grant program to further bolster housing mobility efforts and research [70].

### 4.3. Community Planning and Improvement Strategies

Community planning efforts include community-wide engagement, planning, and investment to address barriers to health and social determinants of health in high-risk neighborhoods. Community improvement strategies are comparable to traditional community development practices but more focused on key social determinants of health and on households that are most marginalized and face the greatest health risks. Community engagement and alignment of diverse institutional and community stakeholders are central to community improvement strategies. Community planning efforts are ideally structured in a way that conforms to Warner and Zhang’s (2020) factors that support healthy places for children, including multi-sector cross agency collaboration, robust engagement with families and youth, and development of a common vision that respects the dimensions of age, race, and ethnicity [71].

Focused neighborhood-based housing strategies to support health equity, led by health stakeholders, are rare but not unprecedented. For example, the Healthy Neighborhoods Healthy Families initiative run by Nationwide Children’s Hospital in Columbus, OH, has led to more than $70 million in investment in the past decade in the hospital’s home neighborhood (Yen et al., 2018). Much of this investment has sought to stabilize various forms of affordable housing and has reduced residential vacancy rates in the neighborhood from 25% in 2011 to 6% in 2016 [72].

The Northern Manhattan Perinatal Partnership (NMPP) was one of the earliest coordinated community improvement efforts focused on addressing disparities in infant and maternal health. A complex and long-term collaboration with Head Start aligned multiple sectors to engage, plan, and support maternal health in the Harlem neighborhood of Northern Manhattan [73]. The program focused on the development of a prenatal assistance program and of a culturally competent peri-natal network to serve the community and on programs to reduce the risks of domestic violence, substance abuse, and housing instability [74]. During the implementation of the NMPP, the program served more than 10,000 mothers, and infant mortality rates in the neighborhoods served by the initiative declined from 27.7 infant deaths per 1000 live births in 1990 to 8.1 in 2008 [75].

Building from the model of the NMPP, the Better Baby Zone (BBZ) model would attempt to pilot a place-based community planning approach to supporting infant and maternal health in multiple cities throughout the United States. As described by Pies et al. (2016), the BBZ seeks to engage communities, align stakeholders, and address critical life stressors at a neighborhood scale (such as housing) [76].

“Best Babies Zone (BBZ) is an early attempt to put life course theory into practice, taking a place-based approach to reducing infant mortality by aligning resources, building community leadership, and transforming educational opportunities, economic development, and community systems in concentrated neighborhoods…each BBZ crafted resident-driven strategies for decreasing the root causes of toxic stress and poor birth out-comes”

[76] (p. 68).

The BBZ model was financially supported by the W.K. Kellogg Foundation and piloted in three cities, with an additional six cities slated for expansion in the future [74]. Only preliminary evaluations of the BBZ pilot initiatives have been done, with most evaluation outcomes at this time limited to community engagement, establishment of new community programming, and partnership development [76,77].

Although the Best Baby Zone model has not produced data to quantify changes in infant mortality, a similar programmatic intervention in Columbus OH (Celebrate One) has demonstrated positive results. Celebrate One focused on a multi-sector community improvement approach in 13 neighborhoods (zip codes) that had the highest infant mortality rates in Franklin County, OH. Since the program’s inception, the Black infant mortality in the focus areas has declined from 19.0 infant deaths per 1000 live births in 2011 to 13.4 infant deaths per 1000 live births in 2019 [78]. The program directly addressed housing needs through the Healthy Beginnings at Home initiative, which focused on stabilizing housing and providing wrap-around services for approximately 50 high-risk first- or second-trimester expectant mothers in the focus neighborhoods [79]. The mothers have maintained stable housing for the past two years, although recent economic impacts of the coronavirus disease 2019 (COVID-19) pandemic threatens to destabilize families [79].

### 4.4. Programmatic Challenges Facing Housing Interventions for Maternal and Infant Health

Evaluations of various housing interventions to support infant and maternal health have identified several structural challenges inhibiting these potential interventions. A national evaluation of Best Baby Zone programs noted that initiatives run by Public Health agencies have difficulty abandoning traditional “tried and true” public health interventions, which are easier to implement and more familiar to public health agencies. The evaluation noted that even within the Best Baby Zone model, the traditional Maternal and Child Health activities (such as health service fairs and wellness services) were often misdirected to mothers which severe economic hardship. As described by Pies et al. in 2016: “women in Hollygrove (a Best Baby Zone pilot site) had not expressed concern about the accessibility of health services, but rather about a lack of reliable transportation, financial issues, underemployment, and inadequate housing as major stressors” [76] (p. 972). Pies et al. (2018) suggest that public health agencies need to better design interventions around community engagement efforts that bring the most critical needs of maternal and child health populations to the forefront [76].

Families experiencing high levels of chronic stress and trauma can present complex cases that can also overwhelm public health practitioners or affordable housing providers [80]. A complex web of factors can influence the ability to secure safe, stable, and affordable housing. Financial barriers, legal barriers, housing market dynamics, labor market dynamics, behavioral and mental health challenges, and family violence can all impact housing stability for lower income renters. Evaluation of the Celebrate One initiative notes the complex set of challenges each family presented in the context of housing needs: “each family came…with a unique constellation of needs and strengths, many shouldering the weight of trauma and deep poverty. At baseline, mothers had many barriers to housing stability, such as having no credit score (54%), a criminal record (44%) or no income (46%). Many also had behavioral health conditions and were experiencing intimate partner violence.” [79] (p. 5). A robust integration of trauma-informed community development practice could assist in meeting the complex needs of households and communities that are the focus of housing interventions to support maternal and child health [80].

## 5. Conclusions

Poor infant health outcomes not only are an indicator of broader societal health and wellbeing but also contribute greatly to long-term health care costs. Stable, healthy, and affordable housing is critical to maternal health and child development and can be fundamental in providing an intervention to address a key social determinant of health. As demonstrated in the preceding literature review, the evidence documenting the significance of housing as both a contemporary and a historical influence for infant and maternal health is rich and compelling.

Public health was a primary motivation for the emergence of early housing strategies such as building codes, early zoning, nuisance laws, slum clearance, and public housing. Despite this rich history, contemporary policymaking in affordable housing and health care or public health remains highly siloed, although collaboration between different fields has grown stronger in recent decades. As demonstrated in the preceding literature review, the evidence documenting the significance of housing as both a contemporary and a historical influence for infant and maternal health is rich and compelling. Given the abundant literature documenting the importance of housing to infant and maternal health, why are the interventions directed to meet the needs of mothers with young children not more widespread (particularly, interventions focused on families that are Black Indigenous People of Color (BIPOC))? I conclude exploring larger systemic challenges which undermine these collaborative solutions.

### 5.1. Housing as a Historical and Contemporary Social Determinant of Maternal Health

Housing is a complex social determinant of health, in that it is historically informed by decades of discriminatory policy in U.S. metropolitan areas. For much of the 20th century, discriminatory housing policy in the United States fostered segregation, limited housing choices for racial and ethnic groups and lower income communities and spurred long-standing disinvestment in urban communities of color. These historical conditions set a foundation for contemporary housing challenges such as housing instability, exposure to poor-quality housing, and isolation into distressed neighborhood environments. These contemporary conditions create increased health risks, such as increased exposure to toxins, chronic stress from housing instability/cost, and environmental stressors. These structural conditions, which are the manifestation of historical discrimination, contribute to the disparities in birth outcomes for mothers and infants, particularly for women of color.

### 5.2. Housing as a Mutually Reinforcing Stressor and Manifestation of Structural Racism Impacting BIPOC Mothers and Children

It is critical to acknowledge that, as a multi-dimensional risk factor, the various forms of housing stress identified previously (housing condition, instability/affordability, and neighborhood effect) are also overlapping and mutually reinforcing risk factors. In many circumstances, the same household is subjected to two or all three of these detrimental housing factors, representing a form of cumulative health risk. In many cases, the most housing-instable, cost-burdened mothers living in the poorest quality housing also reside in the most challenging neighborhood environments. This cumulative risk is a form of cumulative discrimination or disadvantage [81], placing mothers and infants living in these circumstances at significant risk for poor health outcomes. More specifically, housing is deeply implicated as a manifestation of structural racism impacting mothers and children who are BIPOC. Housing disparities are built upon both historical and contemporary forms of racial discrimination. Housing is a critical domain that situates families within a complex web of cumulative health risk and deprivation. Housing interventions could be instrumental in dismantling structural racism and as a mechanism for supporting racial and health equity.

### 5.3. Housing as a Lever for Health Improvement

Housing presents multifaceted health risks but is also a critical intervention point (or lever) to improve health outcomes. Emergent practices have centered around three forms of housing intervention, i.e., direct targeted assistance, triaging resources, and wrap-around services to high-need populations, housing mobility programs which provide stable housing in high-resource environments, and comprehensive community improvement programs that combine community engagement, housing, and built environment improvements and other health supports in higher risk neighborhoods. Although efforts to align housing interventions with maternal and infant health are emergent, the early outcomes of these interventions provide promising evidence of their potential. Continued collaboration between the public health and the housing and community development sectors creates the potential for leveraging housing interventions to support maternal and infant health and reduce disparities in maternal health outcomes. To be an effective anti-racist intervention, housing and maternal health interventions must be developed through a racial-equity lens and be centered around supporting the agency and voice of BIPOC mothers [82].

### 5.4. Barriers to Expanded Housing and Infant and Maternal Health Collaborations

National scans of housing interventions to support health found that most initiatives rarely produce new affordable housing units. Initiatives are more likely to work within existing affordable housing systems to align existing resources to medically vulnerable populations [65]. The reliance on the existing housing social safety net is undermined by the long-term embrace of neoliberal housing policy in the United States and the more recent austerity cuts to housing assistance. Housing programs to support maternal health are working within a complex and racialized U.S. housing system that does not meet the needs of lower income renters.

According to Harvard’s Joint Center for Housing Studies, almost half (47.4%) of the U.S. renters paid more than 30% of their incomes for rent in 2017 [83]. Almost a quarter of renters (10.7 million households) had a severe rent burden, paying over half their incomes for rent. While homeowners also face housing cost burdens, almost 60% of the nation’s severely cost-burdened households were renters. Due to insufficient funding for assisted rental housing, the vast majority of cost-burdened households who qualify for housing assistance are not receiving federal support. Only 23% of those meeting eligibility requirements for housing assistance receive housing support from the U.S. Department of Housing and Urban Development. The 77% of eligible households that do not receive support represent over 17 million households in the United States [84].

One reason behind high renter cost burdens is the loss of affordable units. The number of low-rent units (renting at less than $800 per month) has declined from more than 60% of the rental housing inventory in 2011 to just more than 40% of total rental housing in 2017, leading to a large gap between the supply and the demand for low-rent units. The largest gap in housing supply is for renters who are extremely low-income (earning less than 30% of the area median income). More than 11 million rental households were extremely low-income in 2017, while only 4 million rental units were affordable to this group in the housing market at that time. Affordability restrictions will expire on more than 1.2 million subsidized rental units by 2029, further exacerbating affordability challenges for low-income renters [83].

The safety net of federal supports for rental assistance and affordable housing has withered due to austerity measures implemented since 2010. Federal spending for rental assistance in the United States declined by 13% between 2010 and 2013 (a funding cut of $6.2 billion). After austerity measures were lifted, federal spending was still 4% lower (representing a loss of more than $2 billion) in 2016 than spending in 2010 [85]. Funding cuts to rental assistance have continued since 2016, and the economic impacts of the COVID-19 pandemic have further exacerbated affordable housing challenges.

In addition to structural challenges to federal affordable housing funding, misalignment and misunderstanding between the housing and the health sectors can also undermine their collaboration [86]. A 2018 essay published in the leading affordable housing blog Shelterforce documented the foundational misunderstandings between the housing and the health fields. As described by Lowe [87], misunderstandings in regard to resources, roles, and capabilities in these new collaborations create substantial barriers to innovation between sectors.

“Housing developers see a health care industry that represents a significant share of the U.S. economy and can’t understand why it isn’t interested in underwriting the cost of resident services staff in the nonprofit affordable housing sector to assure better outcomes. One of the reasons for this misalignment is that health care organizations often assign these partnerships to their marketing departments, which regard the relationships as branding opportunities rather than as significant long-term partnerships to alter the social determinants of health. Those who work in the health care industry don’t understand the limits on a housing organization’s funding, so they are confused when they’re asked to pay for staff the housing organizations appear to already have”

[87] (p. 2).

Chaiyachati et al.’s 2016 paper *Health systems tackling social determinants of health: promises, pitfalls, and opportunities of current policies* identifies three primary mechanisms which could invigorate the role of the health sector in addressing social determinants of health [88]. Federal tax policies which require Community Health Needs Assessments (and action plans) be produced by all non-profit hospitals, value-based payment reforms, and the Center for Medicaid Services Accountable Health Communities program are identified as recent reforms that could be leveraged to encourage a more direct and better resourced role for the health system in directly remedying social determinants of health. Given the structural resource challenges facing the affordable housing sector in the United States, reforms like these could be instrumental in opening new resources for housing interventions to support infant and maternal health.

Interventions efforts to align housing and maternal and child health can no longer rely upon existing funding models. Health care resources (particularly, Medicaid resources in the U.S. context) could provide new revenue models to expand housing resources to high-need populations. Programmatic interventions that could utilize Medicaid “cost savings” directly attributed to addressing social determinants of health could be a resource to meet housing needs. More importantly, expanded collaboration, relationship building, and shared knowledge between the affordable housing and the health sectors are critical to building a foundation to support cross-sector solutions to meet the housing needs of maternal and child populations.

## Data Availability

Data sharing not applicable.
